# Operationalisation of Mild Cognitive Impairment: A Graphical Approach

**DOI:** 10.1371/journal.pmed.0040304

**Published:** 2007-10-30

**Authors:** Fiona E Matthews, Blossom C. M Stephan, John Bond, Ian McKeith, Carol Brayne on behalf of the Medical Research Council Cognitive Function and Ageing Study

## Abstract

A new online tool allows mapping of the different classifications of mild cognitive impairment.

Despite intensive use of the term mild cognitive impairment (MCI) to describe an intermediate stage of cognitive decline between normal and pathological brain ageing, no formally agreed process of characterising this condition exists [1–3]. Various definitions have been proposed in the literature, each with differences in focus (e.g., age-associated change versus pathological decline) and non-uniform diagnostic criteria [4–18]. The degree of inconsistency is not trivial: current classifications define heterogeneous populations with different patterns of aetiology, cognitive decline, and clinical outcome [19].

As an opportunity for identifying individuals at risk of developing dementia, MCI is an important concept. Yet lack of consensus criteria has lead to debate about the utility of MCI, resulting in calls for abandoning its diagnosis and adopting an alternative nosology [20–22]. Consensus conferences are now being held, even though MCI diagnoses are already used in clinical trials for prevention of Alzheimer disease [23]. The aim of this paper is to develop a framework for mapping the different classifications of MCI using retrospective information, assessing variations in defining criteria.

## Creating a Framework for Mapping MCI

The first step to coding MCI is to determine the necessary criteria and thresholds for operationalisation of each definition. We compiled a comprehensive list of those classifications which represent different aspects and definitions of MCI. The necessary components for each were abstracted and formulated into a diagnostic algorithm.

The main problem encountered was that while some classifications have specific criteria for implementation (e.g., amnestic MCI [A-MCI] [13,14] and age-associated memory impairment [5]), others are vague descriptions that require interpretation as to the exact nature of the deficit (e.g., age-related cognitive decline [24]). Further complicating the problem is a lack of specification of screening tools and variability in: (1) the domain of impairment (memory versus non-memory, single- versus multi-domain deficits); (2) cut-off scores; (3) acceptable restriction on activities of daily living; and (4) exclusion criteria.

**Figure 1:**
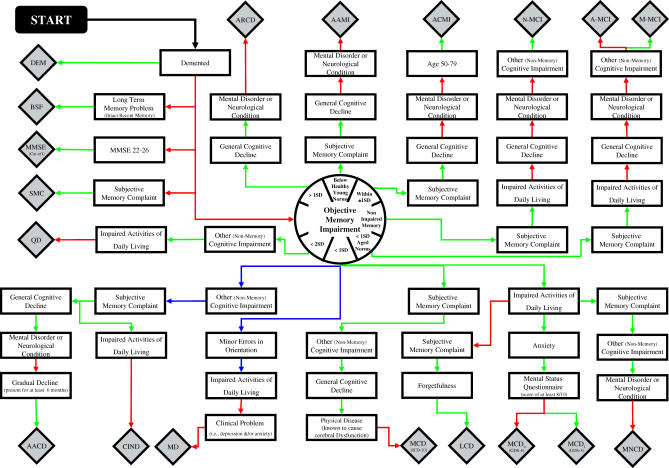
Schematic Overview of the Approach Used to Classify MCI across Different Definitions in the Medical Research Council Cognitive Function and Ageing Study AACD, age-associated cognitive decline; AAMI, age-associated memory impairment; ACMI, age-consistent memory impairment; ARCD, age-related cognitive decline; CIND, cognitive impairment no dementia; DEM, dementia; LCD, limited cognitive disturbance; MCD (ICD-10), mild cognitive disorder (International Classification of Diseases 10th Revision); MCD_i_ (GDS3), mild cognitive decline (Global Deterioration Scale Stage 3); MCD_o_(GDS4), moderate cognitive decline (Global Deterioration Scale Stage 4); MD, minimal dementia; M-MCI, multiple mild cognitive impairment; MMSE, Mini Mental State Examination Cut-off Scores; MNCD, mild neurocognitive disorder; N-MCI, non-amnestic mild cognitive impairment; QD, questionable dementia; SD, standard deviation; SMC, self-reported memory complaint.

Eighteen current definitions of early cognitive impairment were identified in a systematic review of the literature and mapped using a flow diagram as shown in Figure 1. Mapping is completed in two phases: following exclusion of all individuals with dementia, each classification is then operationalised independently. Each classification could be constructed from a subset of 15 different criteria, with memory impairment required as an essential feature in almost all classifications. Surprisingly, no two classification systems map on the same path. Each classification is operationalised in the population and has been previously applied to the Medical Research Council Cognitive Function and Ageing Study (MRC CFAS) [19]. Table 1 outlines how each set of criteria was operationalised in CFAS. To facilitate cross classification comparisons, criteria were consistent in terms of level of impairment unless cut-off thresholds were uniquely specified.

**Table 1:**
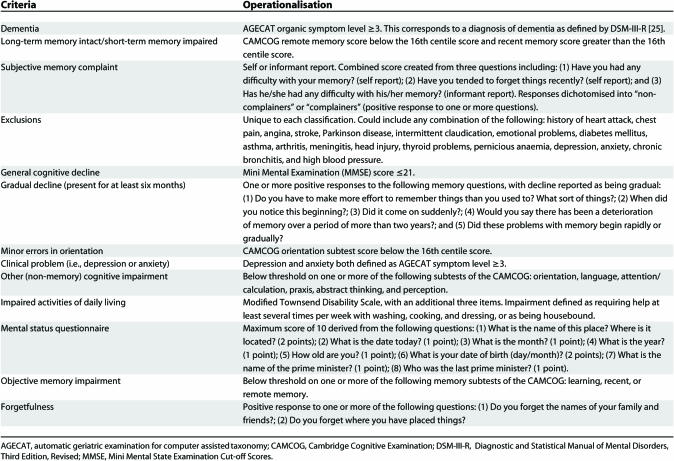
Operationalisation of Components of Classification Systems in the Medical Research Council Cognitive Function and Ageing Study

In reading the flow diagram, the classification arrived at depends on the direction of decision at each criteria (*yes,* criteria required are fulfilled as indicated by a green arrow, or *no,* criteria are not fulfilled as indicated by a red arrow). If an individual fails to meet the specified outcome for a given criterion they are excluded from further mapping.

## Two Examples: Benign Senescent Forgetfulness and A-MCI

**Figure 2:**
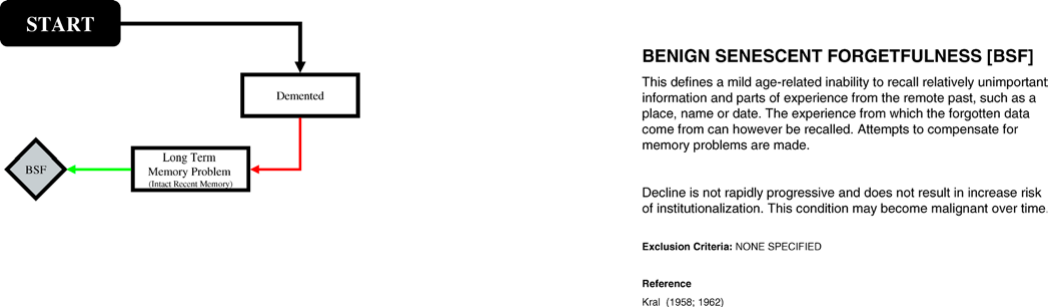
Operationalisation of Benign Senescent Forgetfulness in the Medical Research Council Cognitive Function and Ageing Study

For example, following the flow diagram to arrive at a classification of benign senescent forgetfulness (BSF) takes just three steps: from the *“START”* box you move to the *“Demented”* box. If the individual does not have dementia (as indicated by the red arrow), you next move to the *“Long-Term Memory Problem”* box, and if the individual shows a long-term memory problem with intact recent memory (as indicated by the green arrow), you then arrive at a classification of BSF. (See Figure 2 for the BSF-specific path.)

**Figure 3:**
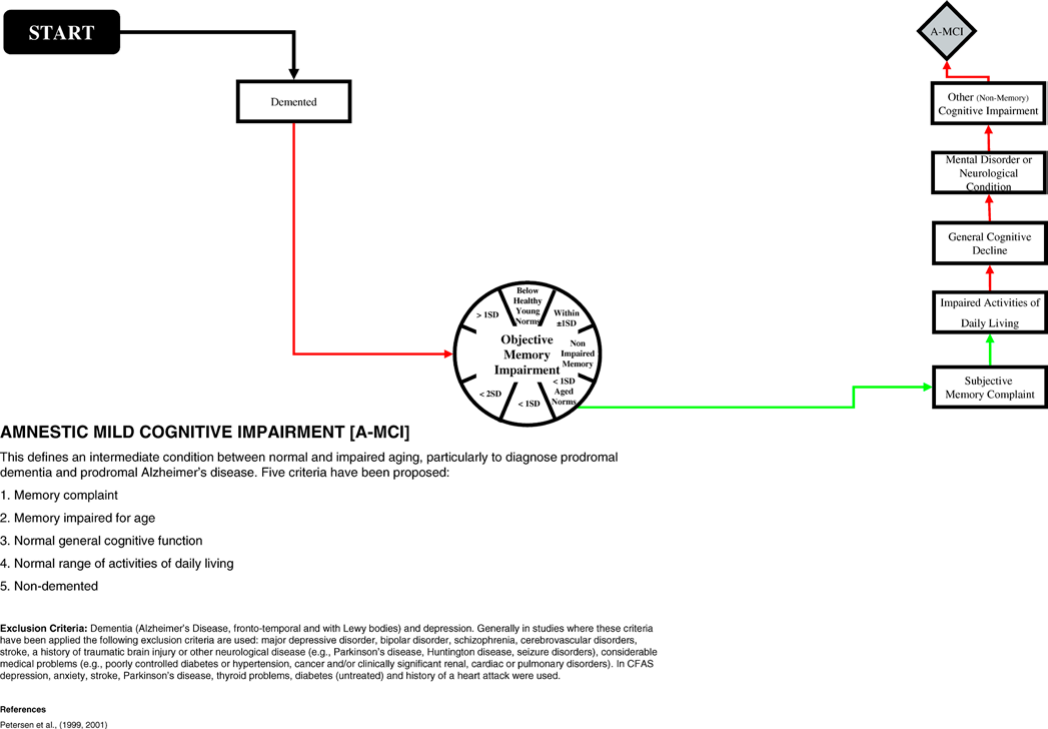
Operationalisation of Amnestic Mild Cognitive Impairment in the Medical Research Council Cognitive Function and Ageing Study SD, standard deviation

In contrast, to arrive at a classification of A-MCI, seven steps are needed: (1) the individual is not demented (as indicated by the red arrow); (2) there is an objective memory impairment (one standard deviation below age-corrected norms, as indicated by the green arrow); (3) the individual or an informant complains of memory loss (as indicated by the green arrow); (4) there is no impairment in activities of daily living (as indicated by the red arrow); (5) there is no impairment of general cognitive functioning (as indicated by the red arrow); (6) the individual has no underlying medical or neurological condition (as indicated by the red arrow); and (7) there is no impairment in another non-memory domain (as indicated by the red arrow). (See Figure 3 for the A-MCI-specific path.)

## Advantages and Disadvantages

The flow diagram details this process for each of the 18 different classification systems, allowing for reproduction across studies. Each concept has been detailed in turn with a criteria-specific graph and the appropriate references on the CFAS Web site (http://www.cfas.ac.uk/mciprogram/). The clinical utility of the concept of mild cognitive impairment depends on the validity of the diagnosis and the ability to predict higher rates of progression to dementia. The advantage of retrospective definition of these concepts is that they are not adjusted by current knowledge or changing criteria, an unfortunate downside of consensus criteria being that they can be influenced by changing knowledge over time. The disadvantage of mapping with retrospective information is that it removes clinical experience from the definition, and the information measured from all individuals must have enough scope to encompass the entire original definition.

A fundamental problem with a multiple system approach is the failure in consistency of classification. Prevalence estimates for each classification have been previously calculated in CFAS using the pathways shown in Figure 1, and were found to be highly variable (range 0.1%–42%) [19]. Some of this difference results from the fact that not all criteria explain pathological ageing, but rather “normal” ageing. Although the distinction between those definitions associated with normal age-related change and those with pathological ageing is not apparent from prevalence estimates alone, it is seen with lower conversion to dementia in those groups defined by non-pathological classifications. Furthermore, the same individual could be classified as impaired on one system and normal on another, even within criteria that are supposedly investigating abnormal change. This makes the interpretation and comparison of results across studies very difficult, where not only the populations but additionally the criteria chosen to estimate MCI are different.

At first glance, a solution to the complexity of the diagram appears simple: reduce all classification systems to a single concept through the amalgamation of all defining criteria, particularly the measurement of objective memory and the medical (and disability) exclusion criteria. However, this solution assumes that within these criteria there is one that is the best for identification of at-risk individuals. Furthermore, definitions, particularly those of objective cognitive impairment, depend on arbitrary and varying thresholds, frequently with no reference to specific values, methods, or screening measures. In retrospective studies, the mapping of these thresholds will primarily be constrained by study design, though the use of different thresholds can be used to determine the most optimal threshold value to accurately distinguish those individuals at high risk of dementia from those with low dementia risk.

## Conclusion

It is time to re-examine the concept of MCI. The diagnostic disparity and the lack of consistency in case definition calls into question what exactly is being captured in each classification. This is a fundamental weakness of research on MCI, as highlighted by the complicated nature of Figure 1. Using this flow diagram, MCI systems can be mapped in other population datasets to investigate: (1) what are the best boundaries for impairment; (2) which tests are most sensitive for measuring each criteria; (3) which criteria, if any, can adequately predict individuals at risk of developing dementia; and (4) would adopting multiple systems across different populations (specialist clinic versus population based) and age groups be more appropriate? It is hoped that graphical operationalisation of the criteria will aid in diagnostic consistency and assist in the visualisation of the current problem, with the aim of formulating a gold standard definition for both research and clinical practice.

## Abbreviations

A-MCI: amnestic mild cognitive impairment
BSF: benign senescent forgetfulness
MCI: mild cognitive impairment
MRC CFAS: Medical Research Council Cognitive Function and Ageing Study
